# Supplementary material for: Double-Chain Cationic Surfactants: Swelling, Structure, Phase Transitions and Additive Effects
Source: Molecules. 2021 Jun 28;26(13):3946. doi: 10.3390/molecules26133946 (PMC8271693; doi:10.3390/molecules26133946)
Supplement: Supplementary file 1 [file molecules-26-03946-s001.zip › molecules-1237418-supplementary.pdf]

Supplementary Materials

Double-chain cationic surfactants: Swelling, structure, phase transitions and additive effects

Rui A. Gonçalves [1], Yeng-Ming Lam [1,*] and Björn Lindman [1,2,3*]

[1] School of Materials Science and Engineering, Nanyang Technological University, 639798 Singapore, Singapore; rui.goncalves@ntu.edu.sg (R.A.G.); ymlam@ntu.edu.sg (Y.-M. L.)

[2] Division of Physical Chemistry, Department of Chemistry, Centre for Chemistry and Chemical Engineering, Lund University, SE-221 00 Lund, Sweden; bjorn.lindman@fkem1.lu.se (B.L.)

[3] Coimbra Chemistry Center (CQC), Faculty of Sciences and Technology, University of Coimbra, 3004-535 Coimbra, Portugal

* Correspondence: ymlam@ntu.edu.sg (Y.-M. L.); bjorn.lindman@fkem1.lu.se (B.L.)

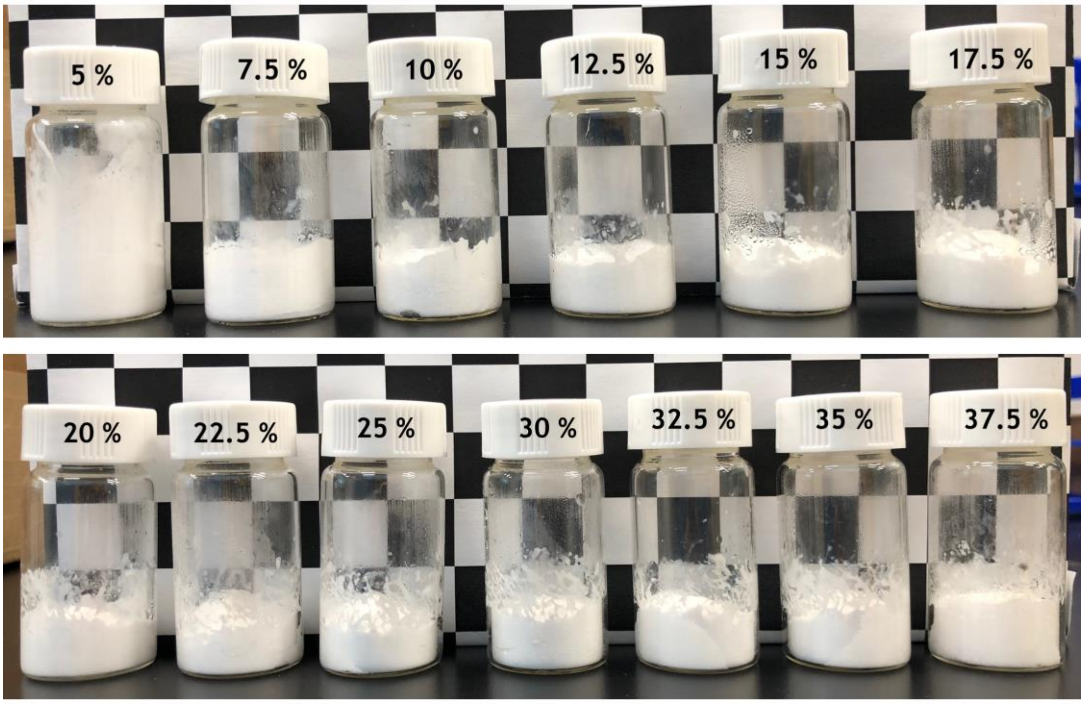

Figure S1. Photographs of the DODAB-water gels from 5-37.5 wt% below the Krafft temperature.

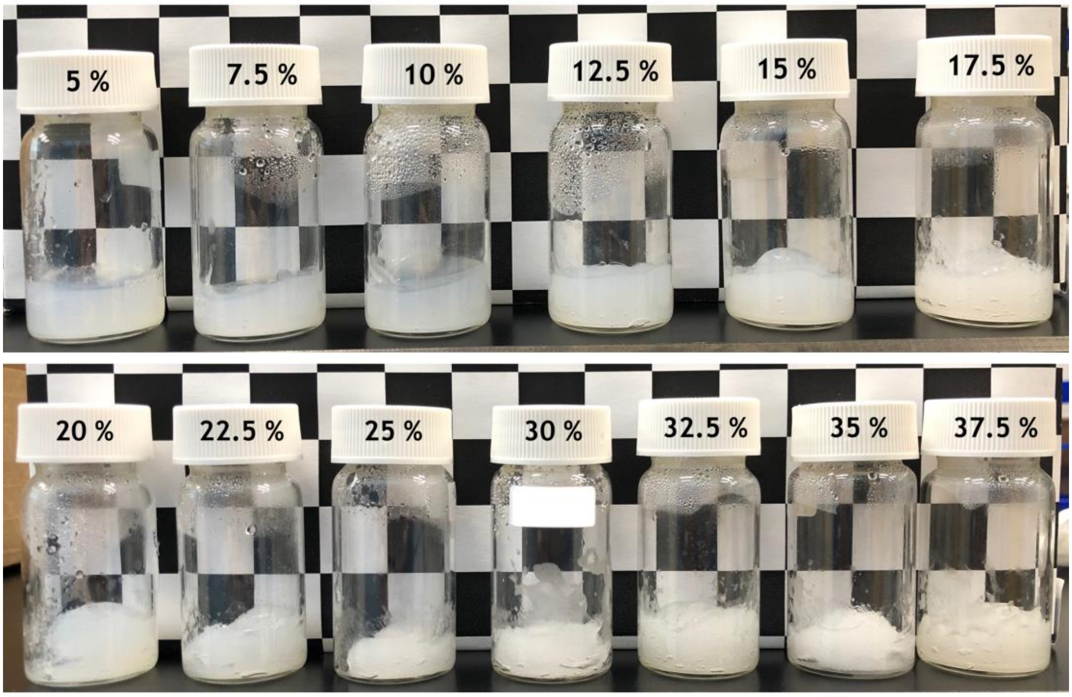

Figure S2. Photographs of the DODAC-water gels from 5-37.5 wt% below the Krafft temperature.

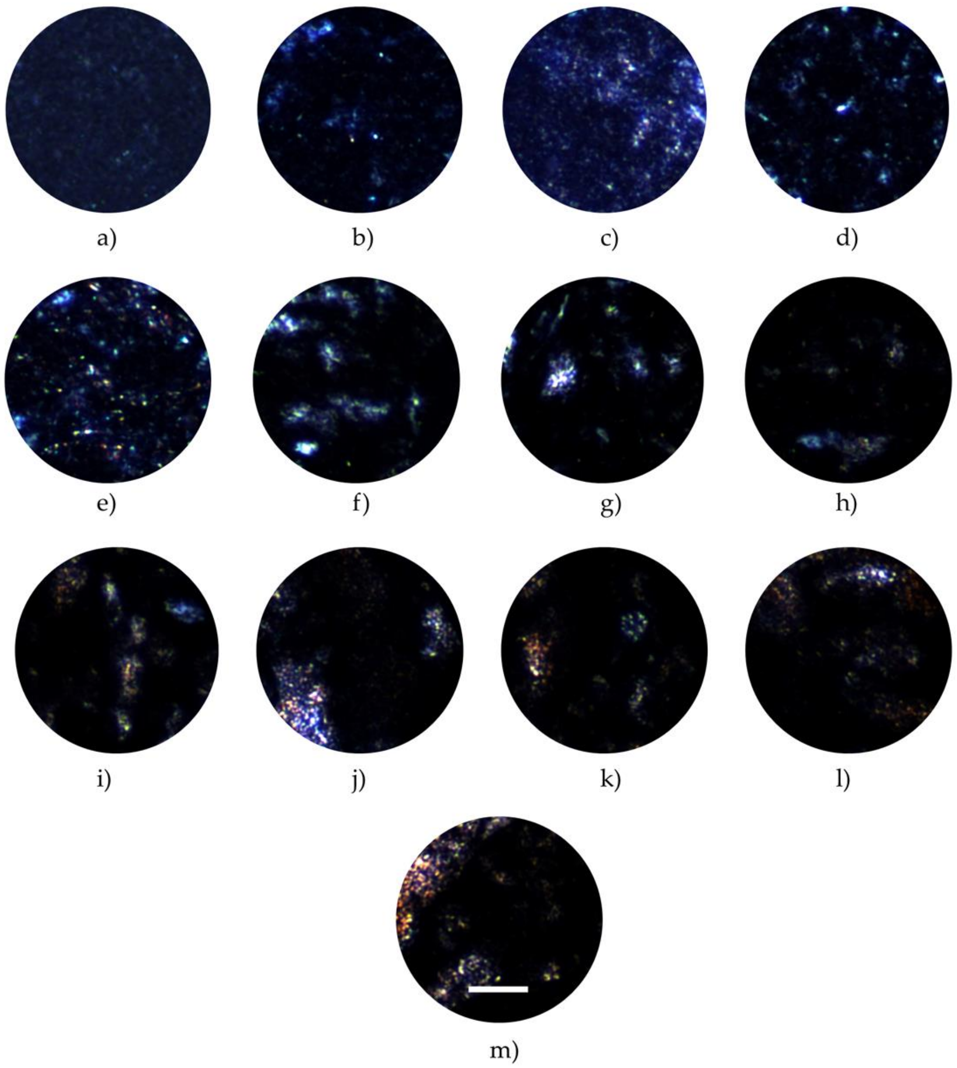

Figure S3. POM micrographs of the textures of DODAB in water at increasing surfactant concentration: (a) to (m) correspond to 5 wt%, 7.5 wt%, 10 wt%, 12.5 wt%, 15 wt%, 17.5 wt%, 20 wt%, 22.5 wt%, 25 wt%, 30 wt%, 32.5 wt%, 35 wt% and 37.5 wt%, respectively. The scale was set at 50 μm and applied to all micrographs. Temperature was kept constant at 25 ^{o}C.

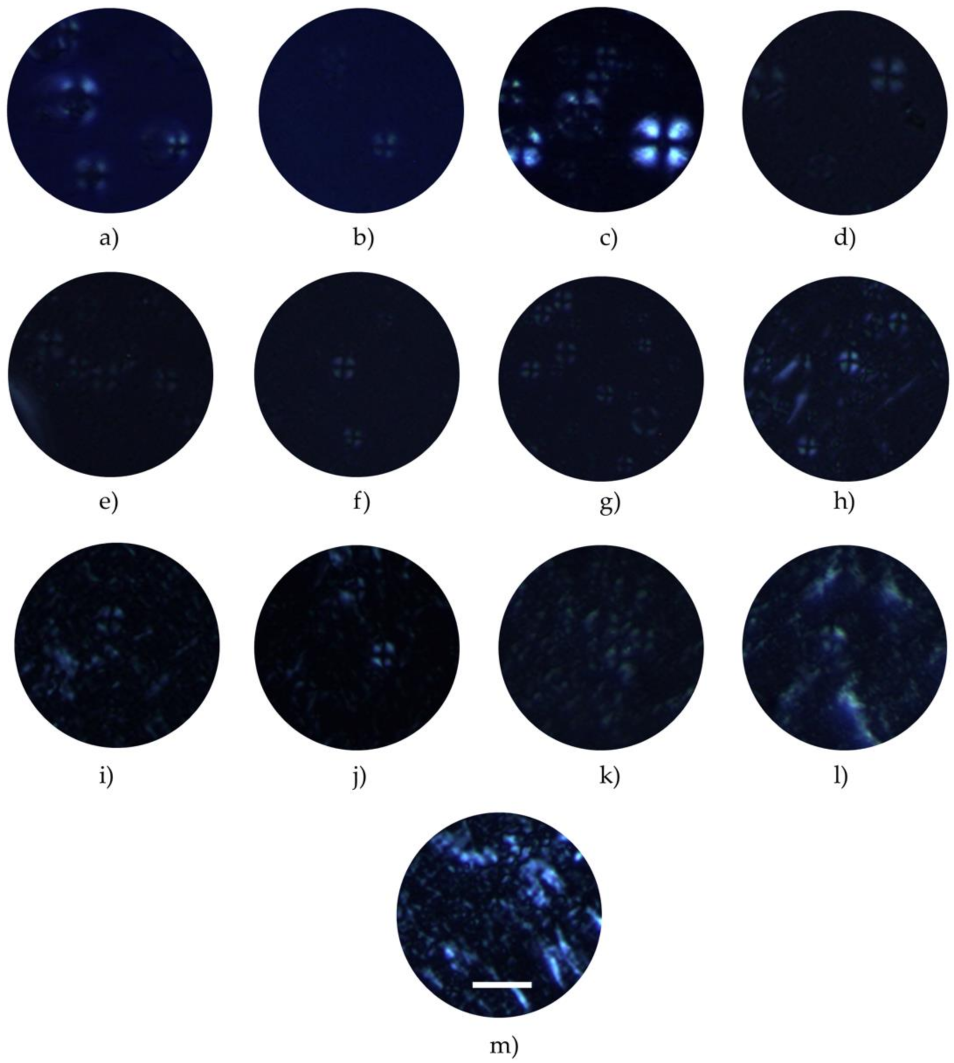

Figure S4. POM micrographs of the textures of DODAC in water at increasing surfactant concentration: (a) to (m) correspond to 5 wt%, 7.5 wt%, 10 wt%, 12.5 wt%, 15 wt%, 17.5 wt%, 20 wt%, 22.5 wt%, 25 wt%, 30 wt%, 32.5 wt%, 35 wt% and 37.5 wt%, respectively. The scale was set at 50 μm and applied to all micrographs. Temperature was kept constant at 25 ^{o}C.

Effects of the DSC scanning rate on the Tm of the surfactant.

To understand the implications of the scan rate in the determination of the thermodynamic parameters associated to the phase transformations of the surfactant systems in water, a study of the thermal behavior at four different scan rates was performed. It has been previously discussed in literature that the sharp peak due to the first-order L_β-L_α phase transition of high purity double-tailed surfactant is affected by the scan rate and slow scan rates were recommended to avoid peak broadening [1]. In the case of lamellar surfactant systems, usually when the system was composed of vesicles, they showed to be sensitive to this parameter at low concentrations of surfactant [2]. Therefore, understanding the effect of scan rates was crucial to the identification of the thermal transitions present in the system. Our studies focused on systems with high concentration (35 wt%) of surfactant which is seldom discussed in literature and DSC scans (both heating and cooling) are collected at various scan rates - 1, 2, 5 and 10 °C/min to investigate how the scan rate could affect the thermodynamic properties of DODAC in water at high concentration regime.

Figure S5 shows the thermograms obtained under four different scan rates for 35 wt% of DODAC in water. The sample was heated from 10 to 60 ^{o}C (Figure S5a), followed by cooling from 60

to 10 °C (Figure S5b), and the complete heating/cooling cycle can be obtained. The downward peaks of the thermograms indicate an endothermic event. By integrating of the area under or above the peak, the thermodynamic parameters associated with the thermal transitions were obtained, as summarized in Table S1.

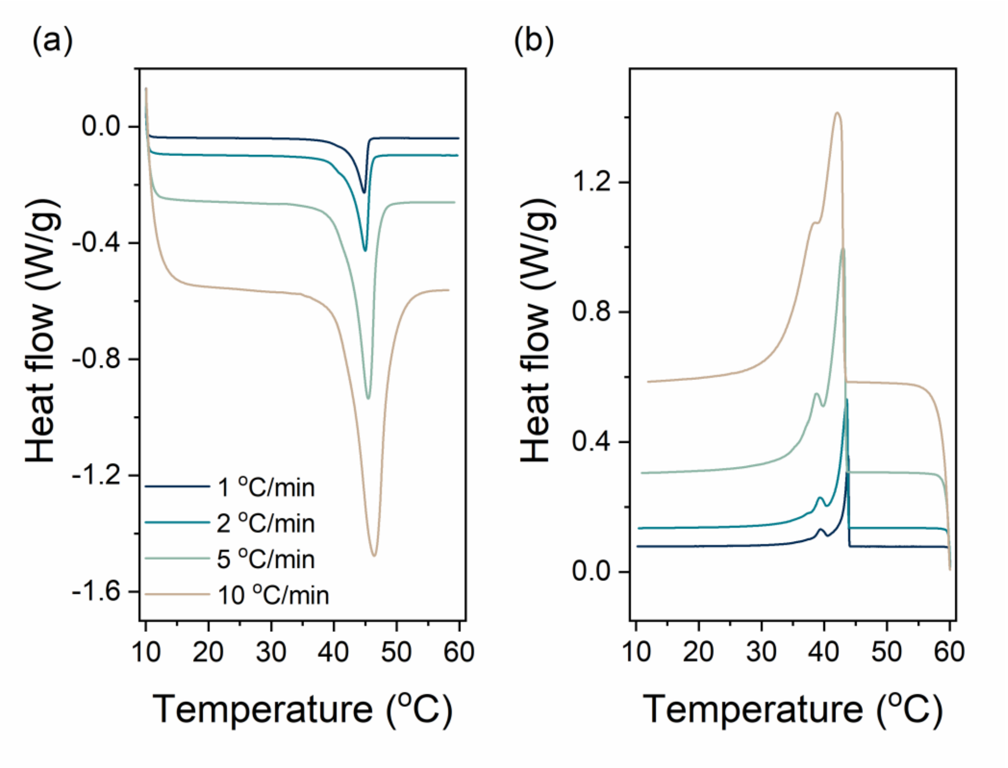

Figure S5. Thermograms under (a) heating and (b) cooling of 35 wt% DODAC in water at four different scan rates (1, 2, 5, and 10 °C /min). The endothermic event corresponds to the downwards deflection.

Table S1. Normalized thermodynamic parameters of 35 wt% DODAC in water under different scan rates.

	**Heating**		**Cooling**	
Scan rate	**T_m (°C)**	**ΔH_m (kJ mol^{-1})**	**T_c (°C)**	**ΔH_c (kJ mol^{-1})**
1 °C/min	44.8	49.2	43.8	50.7
2 °C/min	45.0	49.5	43.6	51.4
5 °C/min	45.4	49.9	43.0	53.8
10 °C/min	46.4	50.4	42.1	53.5

The results show that different thermal scan rates did not affect the shape of the thermograms, both under heating and cooling conditions. One peak was detected during heating, whereas two thermal transition peaks were recorded during cooling, indicating a possible existence of a metastable phase not detected during the heating process. Negligible differences in the T_m and ΔH_m values with increasing scan rates were observed, and a similar trend was found during the cooling scan. An average T_m of 45.4 °C with an average associated ΔH_m of 49.8 kJ mol^{-1} under heating scan were determined. Although scan rates do not seem to have an effect on the temperature and enthalpy associated with the phase transitions, other parameters such as the cooperativity of phase transition may have been affected. As the studies involved high concentration solutions (35 wt%) of double-tailed surfactant, due to pure geometrical constraints between the polar head-group and the hydrophobic tails, such systems are expected to self-assemble into planar lamellar structures. Upon heating, the alkyl chains of one surfactant molecule will start melting and this event is propagated to the neighboring chains, which ultimately results in a lamellar system with the chains in a "fluid-like" state. The cooperativity of the phase transition was shown by the sharpness of the peaks. A sharper and narrower peak is observed

when a slow scan rate was used (Figure S5) demonstrating that even if different scan rates did not significantly affect the T_m and ΔH_m values, it had affected the cooperativity of the alkyl chain melting process. It was verified that at fast scan rates, the phase transformation took place over longer times, thus, resulting in peak broadening. Therefore, the use of slow thermal scan rates to track the phase transformations of the surfactant system with temperature is preferred.

Though the original intention was to investigate the effect of the scan rate on the thermally induced phase transitions for a high surfactant concentration solution (35 wt% of DODAC in water), it was found that the cooperativity of thermal transition was enhanced when the DSC scan was performed at slow scan rates. No significant differences were observed between scans at 1 or 2 ^oC/min thus, the thermal characterization of the surfactant samples was carried out at a scan rate of 2 ^oC/min.

Table S2. Values for the main phase transition temperature (T_m) and the normalized enthalpy of endothermic transition (ΔH_m) for DODAC-*additive*-water ternary systems.

	**Composition (wt%)**		**Molar ratio**	**T_m**	**ΔH_m**
	**DODAC**	**additive**	**(S:A:W)**	**(^oC)**	**(kJ mol^{-1})**
DODAC in water	35.0	0.0	1:0.0:60	45.1	45.8
Urea	35.0	5.0	1:1.4:56	45.8	42.6
	35.0	7.5	1:2.1:53	46.5	42.0
	35.0	10.0	1:2.8:51	45.4	46.8
	35.0	12.5	1:3.5:49	45.3	39.8
	35.0	15.0	1:4.2:47	45.5	43.7
	35.0	20.0	1:5.6:42	45.7	44.7
Methyl urea	35.0	5.0	1:1.1:56	45.8	41.7
	35.0	7.5	1:1.7:53	45.6	37.5
	35.0	10.0	1:2.3:51	46.2	37.4
	35.0	12.5	1:2.8:49	45.9	36.7
	35.0	15.0	1:3.4:47	46.0	44.2
	35.0	20.0	1:4.5:42	46.1	39.9
Dimethyl urea	35.0	5.0	1:1.0:56	45.9	41.8
	35.0	7.5	1:1.4:53	45.7	32.1
	35.0	10.0	1:1.9:51	46.0	50.3
	35.0	12.5	1:2.4:49	47.1	35.2
	35.0	15.0	1:2.9:47	45.9	46.7
	35.0	20.0	1:3.8:42	45.6	43.5
Acetic acid	35.0	5.0	1:1.4:56	43.5	44.5
	35.0	7.5	1:2.1:53	42.3	43.6
	35.0	10.0	1:2.8:51	41.5	43.5
	35.0	12.5	1:3.5:49	40.7	42.9
	35.0	15.0	1:4.2:47	39.6	42.7
	35.0	20.0	1:5.6:42	37.4	41.1
Propionic acid	35.0	5.0	1:1.1:56	38.8	37.1
	35.0	7.5	1:1.7:53	36.8	39.8
	35.0	10.0	1:2.3:52	34.3	38.9
	35.0	12.5	1:2.9:49	32.1	40.8
	35.0	15.0	1:3.4:46	30.0	39.9
	35.0	20.0	1:4.5:42	24.7	43.0
Butyric acid	35.0	1.3	1:0.2:60	41.8	37.7
	35.0	2.1	1:0.4:58	40.0	36.2
	35.0	4.5	1:0.8:56	35.4	37.1
	35.0	5.0	1:1.0:56	34.6	31.6
	35.0	7.5	1:1.5:53	30.6	32.0

	35.0	10.0	1:1.9:51	26.5	28.6
	35.0	12.5	1:2.4:49	24.9	25.8
	35.0	15.0	1:2.9:47	21.6	25.1
	35.0	20.0	1:3.8:42	*2	*2
Sodium butyrate	35.0	1.3	1:0.2:60	44.8	41.1
	35.0	2.6	1:0.4:58	44.8	41.5
	35.0	5.0	1:0.8:56	45.0	43.0
	35.0	6.6	1:1.0:56	45.0	40.9
	35.0	7.5	1:1.1:53	44.8	43.8
	35.0	10.0	1:1.5:51	46.0	40.6
	35.0	12.5	1:1.9:49	44.9	41.8
	35.0	15.0	1:2.3:47	44.3	43.5
	35.0	20.0	1:3.1:42	43.2	42.3
Hexanoic acid	35.0	5.0	1:0.7:56	26.4	29.4
	35.0	7.5	1:1.1:54	22.5	24.1
	35.0	10.0	1:1.4:51	21.0	18.5
	35.0	12.5	1:1.8:49	13.4	4.6
	35.0	15.0	1:2.2:47	14.3	*2
	35.0	20.0	1:2.9:42	*1	*1
Benzyl alcohol	35.0	5.0	1:0.9:56	35.5	35.7
	35.0	7.5	1:1.2:53	33.0	34.3
	35.0	10.0	1:1.6:51	29.7	34.5
	35.0	12.5	1:1.9:49	28.2	34.4
	35.0	15.0	1:2.3:47	21.1	31.5
	35.0	20.0	1:3.1:43	18.9	31.4
Phenoxyethanol	35.0	5.0	1:0.6:56	36.4	46.6
	35.0	7.5	1:0.9:53	32.6	35.2
	35.0	10.0	1:1.2:52	24.8	25.7
	35.0	12.5	1:1.5:49	20.7	19.8
	35.0	15.0	1:1.8:46	19.6	19.6
	35.0	20.0	1:2.4:42	17.1	27.1
Butanol	35.0	5.0	1:1.2:56	37.0	43.9
	35.0	7.5	1:1.7:54	32.2	42.9
	35.0	10.0	1:2.3:51	28.1	42.6
	35.0	12.5	1:2.8:49	25.2	37.9
	35.0	15.0	1:3.4:47	22.4	32.2
	35.0	20.0	1:4.6:42	19.7	24.6
Hexanol	35.0	5.0	1:0.8:56	31.9	30.9
	35.0	7.5	1:1.3:54	29.2	22.0
	35.0	10.0	1:1.6:51	24.8	11.1
	35.0	12.5	1:2.1:49	21.5	*2
	35.0	15.0	1:2.5:47	14.6	*2
	35.0	20.0	1:3.3:42	13.3	*2
Octanol	35.0	5.0	1:0.7:56	27.3	*2
	35.0	7.5	1:1.0:54	26.1	*2
	35.0	10.0	1:1.3:51	25.4	*2
	35.0	12.5	1:1.7:50	19.1	*2
	35.0	15.0	1:1.9:47	20.3	*2
	35.0	20.0	1:2.6:42	13.2	*2
Decanol	35.0	5.0	1:0.5:56	39.1	39.7
	35.0	7.5	1:0.8:54	41.6	32.3

	35.0	10.0	1:1.1:51	40.8	31.9
	35.0	12.5	1:1.3:49	41.5	31.2
	35.0	15.0	1:1.6:47	39.5	31.9
	35.0	20.0	1:2.1:42	39.3	*2
Dodecanol	35.0	5.0	1:0.5:56	41.7	55.0
	35.0	7.5	1:0.7:54	51.4	48.3
	35.0	10.0	1:0.9:51	50.6	50.6
	35.0	12.5	1:1.1:49	52.1	54.2
	35.0	15.0	1:1.3:47	52.3	60.5
	35.0	20.0	1:1.8:43	52.1	65.6

*1 Absence of thermal transition.

*2 More than one thermal transition identified.

Table S3. Values for the bilayer volume fraction (Φ_{bi}), the interlamellar spacing (d_{sp}), the bilayer thickness (d_{bi}) and the area per surfactant molecule (a) for DODAC-*fatty alcohols*-water ternary systems below and above the T_m.

**Additive**	**wt% additive**	**Molar ratio (S:A:W)**	**Φ_{bi}**	**Below T_m**			**Above T_m**			**Transition**	**T_m (°C)**
				d_{sp} (Å)	d_{bi} (Å)	a (Å^2)	d_{sp} (Å)	d_{bi} (Å)	a (Å^2)		
	0.0	1:0.0:60	0.349	69	24	85	84	29	70	L_β-L_α	45.1
**Butanol**	5.0	1:1.2:56	0.382	71	27	81	72	28	80	L_β-L_α	37.0
	7.5	1:1.7:54	0.400	67	27	83	67	27	83	L_β-L_α	32.2
	10.0	1:2.3:51	0.419	64	27	82	62	26	85	L_β-L_α	28.1
	12.5	1:2.8:49	0.435	60	26	85	60	26	85	L_β-L_α	25.2
	15.0	1:3.4:47	0.454	75	34	65	-	-	-	-	22.4
	20.0	1:4.6:42	0.490	71	35	63	-	-	-	-	19.7
**Hexanol**	5.0	1:0.8:56	0.392	114	45	53	73	29	83	L_β/Cub-L_α	31.9
	7.5	1:1.3:54	0.415	150	62	38	-	-	-		29.2
	10.0	1:1.6:51	0.436	172	75	32	-	-	-		24.8
	12.5	1:2.1:49	0.457	172	78	30	-	-	-		21.5
	15.0	1:2.5:47	0.480	-	-	-	-	-	-		14.6
**Octanol**	5.0	1:0.7:56	0.395	131	52	48	79	31	79	L_β/Cub-L_α	27.3
	7.5	1:1.0:54	0.417	96	40	62	75	31	79	L_β/Cub-L_α	26.1
	10.0	1:1.3:51	0.441	98	43	57	-	-	-		25.4
	12.5	1:1.7:50	0.459	105	48	51	-	-	-		19.1
	15.0	1:1.9:47	0.485	104	50	49	-	-	-		20.3
	20.0	1:2.6:42	0.530	125	66	37	-	-	-		13.2
**Decanol**	5.0	1:0.5:56	0.395	99	39	66	77	30	85	L_β-L_α	39.1
	7.5	1:0.8:54	0.419	101	42	61	74	31	84	L_β-L_α	41.6
	10.0	1:1.1:51	0.442	90	40	65	84	37	70	L_β-L_α	40.8
	12.5	1:1.3:49	0.465	84	39	66	82	38	68	L_β-L_α	41.5
	15.0	1:1.6:47	0.488	81	39	66	-	-	-	L_β-Hex	39.5
	20.0	1:2.1:42	0.534	75	40	64	-	-	-	L_β-Hex	39.3
**Dodecanol**	5.0	1:0.5:56	0.396	83	33	82	63	25	107	L_β-L_α	41.7
	7.5	1:0.7:54	0.420	94	39	69	67	28	96	L_β-L_α	51.4
	10.0	1:0.9:51	0.443	89	39	69	-	-	-	L_β-Hex	50.6
	12.5	1:1.1:49	0.466	81	38	71	-	-	-	L_β-Hex	52.1
	15.0	1:1.3:47	0.489	79	38	70	-	-	-	L_β-Hex	52.3
	20.0	1:1.8:43	0.534	76	40	67	-	-	-	L_β-Hex	52.1

References

1. Sturtevant, J. M., Biochemical Applications of Differential Scanning Calorimetry. *Annual Review of Physical Chemistry* **1987,** 38, (1), 463-488.
2. Alves, F. R.; Loh, W., Vesicles prepared with the complex salts dioctadecyldimethylammonium polyacrylates. *Journal of Colloid and Interface Science* **2012,** 368, (1), 292-300.
